# Is there a reliable brain morphological signature for migraine?

**DOI:** 10.1186/s10194-020-01158-7

**Published:** 2020-07-11

**Authors:** Hong Zhou Wang, Wan Hua Wang, Hai Cun Shi, Cong Hu Yuan

**Affiliations:** 1 Department of Neurology, Kunshan Hospital, Affiliated to Jiangsu University, Kunshan, People’s Republic of China; 2 Department of Neurology, Affiliated Yancheng Hospital, School of Medicine, Southeast University, Yancheng, People’s Republic of China; 3 Department of Anesthesia and Pain Management, Affiliated Yancheng Hospital, School of Medicine, Southeast University, West Xindu Road 2#, Yancheng, Jiangsu Province 224001 People’s Republic of China

**Keywords:** Migraine, Voxel-based morphometry, Gray matter, Coordinate-based meta-analysis

## Abstract

Voxel-based morphometry (VBM) is a popular non-invasive magnetic resonance imaging technique to investigate brain gray matter (GM) differences between groups. Recently, two VBM studies in migraine have been published in The Journal of Headache and Pain. Reviewing the two and those previous published VBM studies, we found considerable variations of the results. Spatially diverse brain regions with decreased and increased GM alterations and null findings have been reported. It is interesting to know whether there is a reliable brain morphological signature for migraine. Coordinate-based meta-analysis (CBMA) is increasingly used to quantitatively pool individual neuroimaging studies to identify consistent and reliable findings. Several CBMA have been conducted, however, their results were inconsistent. The algorithms for CBMA have evolved and more eligible VBM studies in migraine have been published. We therefore conducted an updated CBMA using the latest algorithms for CBMA, seed-based d mapping with permutation of subject images (SDM-PSI). The present CBMA of 32 VBM studies (41 datasets comprising 1252 patients and 1025 healthy controls) found no evidence of consistent GM alterations in migraine. Sensitivity analysis, subgroup meta-analyses, and meta-regression analyses revealed that the result was robust. This negative result indicates that there is no reliable brain morphological signature for migraine. VBM investigations in migraine remain a heterogeneous field. Many potential confounding factors, such as underpowered sample sizes, variations in demographic and clinical characteristics, and differences in MRI scanners, head coils, scanning parameters, preprocessing procedures, and statistical strategies may cause the inconsistences of the results. Future VBM studies are warranted to enroll well-characterized and homogeneous subtype samples with appropriate sample sizes, comprehensively assess comorbidities and medication status, and use well-validated and standardized imaging protocols and processing and analysis pipelines to produce robust and replicable results in migraine.

## Background

Migraine is a prevalent and disabling neurological disorder [1]. However, its pathophysiology remains poorly understood. Voxel-based morphometry (VBM) is a popular non-invasive magnetic resonance imaging (MRI) technique that enables us to investigate brain gray matter (GM) differences between groups [2]. In 2003, Matharu and colleagues performed the first whole-brain VBM study in migraine and found no significant GM alterations [3]. Since then, numerous VBM studies have been conducted. Recently, two VBM studies in migraine were published in *The Journal of Headache and Pain* [4, 5]. One study by Liu and colleagues showed decreased GM volume in the right supramarginal gyrus and increased GM volume in the right cerebellar crus II in patients with high-frequency migraine relative to healthy controls [5]. The other study by Bonanno and colleagues reported different results and showed distinct patterns of GM abnormalities in migraine patients with and without aura relative to healthy controls [4]. Such results are noteworthy, however, reviewing the two and previous published studies, we found considerable variations of the VBM results. Spatially diverse brain regions with decreased and increased GM and null findings have been reported (Supplementary Table 1). The replicability and generalizability are increasingly concerned in neuroimaging research. It is interesting to know whether there is a reliable brain morphological signature for migraine, which may be of clinical and translational importance.

## Main text

Coordinate-based meta-analysis (CBMA) is a powerful approach to quantitatively pool individual neuroimaging studies to identify consistent and reliable findings [6, 7]. Several CBMA have been conducted to find consistent brain GM abnormalities in patients with migraine [8–11]. The first CBMA published online on March 15, 2015 using the activation likelihood estimation (ALE) approach included five VBM studies and found GM reductions in the middle and inferior frontal cortices in migraine [8]. Published on May 2nd, 2015, Dai et al. conducted the second CBMA using anisotropic effect size version of signed differential mapping (AES-SDM), which included nine VBM studies and showed consistent GM reductions in the posterior insular-opercular regions, prefrontal cortex, and anterior cingulate cortex [9]. Published on January 19, 2017, the third CBMA of eight VBM studies using ALE by Jia and Yu was performed, who found GM reductions in the bilateral inferior frontal gyri, right precentral gyrus, left middle frontal gyrus, and the left cingulate gyrus in migraine [10]. A most recent CBMA using ALE published as a medRxiv preprint on February 20, 2020 by Masson et al. included 27 VBM studies and detected no significant decrease of GM volume in migraine [11]. We noted that these CBMA studies also demonstrated divergent results. Several factors contribute to the divergences. First, the former three CBMA included relatively low numbers of VBM studies. It has been shown that at least 17 experiments in the ALE analysis would achieve reasonable power for statistical analysis [12]. Second, the algorithms for the CBMA have evolved. The old versions of ALE or use of uncorrected statistical threshold would yield spurious results [13]. The revised version of ALE applied in the recent CBMA used strict statistical threshold [11]. Third, differences of the algorithms applied in the AES-SDM and ALE would yield inconsistent results. ALE only included the studies with significant neuroimaging results. In contrast, AES-SDM quantitatively integrated both significant and null findings [14]. Fourth, variations in inclusion and exclusion criteria for the CBMA may also contribute to the inconsistences. According to the recent best-practice guidelines for CBMA, studies that applied region of interest (ROI)-based analysis or small-volume correction (SVC) have to be excluded [6, 7]. Reviewing the studies included in the CBMA by Masson et al., several studies should not be included.

Recently, a new generation algorithms for CBMA, seed-based d mapping with permutation of subject images (SDM-PSI, https://www.sdmproject.com/), has been presented and recommended [15, 16]. SDM-PSI makes significant improvements, such as use of standard voxelwise tests, standard permutation of subject images (PSI), unbiased estimation of effect sizes based on MetaNSUE algorithms, random-effects models, Freedman-Lane-based permutations, and threshold-free cluster enhancement (TFCE) statistics. These improvements avoid the drawbacks of the alternative procedures used in other current CBMA methods, thus making the results more accurate [15]. We therefore conducted an updated CBMA of available whole-brain VBM studies using SDM-PSI. After a comprehensive and careful literature search and screen, our CBMA finally included 32 whole-brain VBM studies (41 datasets) [3–5, 11, 17–44] involving a total of 1252 patients with migraine (988 females/264males, mean age 37.63 years) and 1025 healthy controls (794 females/231males, mean age 36.78 years). The present CBMA using SDM-PSI did not find evidence of consistent GM alterations in migraine (threshold-free cluster enhancement corrected, *p* < 0.05 and cluster size ≥10 voxels). Complementary analyses, such as sensitivity analysis, subgroup meta-analyses, and meta-regression analyses confirm the result robust. The details of the search strategy, study selection criteria, methods, the Tables regarding the demographic and clinical characteristics (Supplementary Table 1), imaging methodological information (Supplementary Table 2), and results of the SDM-PSI meta-analysis were provided in the Supplementary Materials.

The latest comprehensive CBMA with the largest number of whole-brain VBM studies reveals that there is no a brain morphological signature for migraine. Migraine is a heterogeneous disorder. VBM studies have shown that GM alterations are associated with attack frequency [5, 30, 35, 40], disease duration [21, 35], disease severity [23], aura [4], migraine cycle (during or between attacks) [20], long-term outcomes [5], the number of tablets taken per month [19], and medication overuse [24]. In addition, age and female gender are potential confounding factors that may affect GM alterations in migraine [9, 45–47]. Migraine is associated with higher rates of psychiatric disorders, such as major depression, bipolar disorder, and anxiety disorders [48]. However, these comorbidities have not been comprehensively assessed and controlled in previous VBM studies. In addition, sample sizes in most of the individual VBM datasets were under 50 participants per group. Underpowered studies with small sample sizes may produce unreliable results [49]. A software tool, called PowerMap has been developed to undertake statistical power calculation in neuroimaging studies [50]. Furthermore, it has been proposed that differences in MRI scanners, head coils, scanning parameters, preprocessing procedures, and statistical strategies may yield divergent results in individual VBM studies [49, 51]. The inconsistences prevent us to obtain robust brain morphological features and limit the transdiagnostic effect in migraine. Lately, a more reliable technique called surface-based morphometry (SBM) was introduced to study groups’ cortical thickness differences, which may provide more insights regarding brain morphology for migraine.

## Conclusions

VBM investigations in migraine remain a heterogeneous field. The CMBA of available VBM studies found no evidence of consistent GM alterations in migraine. The quantitative evidence reveals that there is no brain morphological signature for migraine. The search for neuroimaging biomarkers for migraine is still on the way. Future VBM studies call for the control of potential confounding factors, such as the enrollment of well-characterized and homogeneous subtype samples with appropriate sample sizes, comprehensive assessment of comorbidities and medication status, and use of well-validated and standardized imaging protocols and processing and analysis pipelines (use of high field strength MRI with high spatial resolution, more recent software package, appropriate covariates in the statistical model, such as total intracranial volume or total GM volume, age, gender, and other comorbidity, and corrected thresholds for multiple comparisons) to produce robust and replicable results in migraine.

## Supplementary information

**Additional file 1.**

## Data Availability

The datasets generated and/or analyzed during the current study are available from the corresponding author on reasonable request.

## Abbreviations

AES-SDM: Anisotropic effect size version of signed differential mapping
ALE: Activation likelihood estimation
CBMA: Coordinate-based meta-analysis
GM: Gray matter
MRI: Magnetic resonance imaging
ROI: Region of interest
SDM-PSI: Seed-based d mapping with permutation of subject images
SVC: Small-volume correction
VBM: Voxel-based morphometry
