# Supplementary material for: Is there a reliable brain morphological signature for migraine?
Source: J Headache Pain. 2020 Jul 11;21(1):89. doi: 10.1186/s10194-020-01158-7 (PMC7353790; doi:10.1186/s10194-020-01158-7)
Supplement: Supplementary file 1 — Additional file 1. [file 10194_2020_1158_MOESM1_ESM.docx]

**Supplementary Materials**

**Supplementary Methods**

The present meta-analysis was undertaken following the recent guidelines and recommendations for coordinate-based meta-analysis (CBMA) [1, 2].

**Search strategy**

We comprehensively searched several electronic databases: PubMed, Web of Science, and Embase using the keywords (“voxel-based morphometry” OR “vbm” OR “gray matter” OR “grey matter” OR “voxel*”) AND “migraine” up to May 22, 2020. Reference lists of included studies and relevant reviews identified in the search were manually reviewed.

**Study selection criteria**

Included studies had to: (1) investigate patients with migraine according to established criteria; (2) be published as original articles in English; (3) include a headache-free control group for comparison; (4) use a voxel-based morphometry (VBM) method to estimate gray matter (GM) volume or density differences at the whole-brain level between patients with migraine and headache-free controls; (5) provide three-dimensional coordinates of significant clusters in Montreal Neurological Institute [MNI] or Talairach space or report null findings.

The publications were excluded if: (1) they were review articles, meta-analysis, conference abstracts, research protocols, editorials, letters, and commentaries. (2) they enrolled less than seven participants in either the patient group or the control group [2]; (3) they enrolled vestibular or pediatric migraineurs in the study; (4) they did not use a control group; (5) they did not provide peak coordinates of significant clusters; (6) they used region-of-interest analysis or small-volume correction analysis.

In case of overlapping samples, only the study with a larger sample size was selected. In case of results from both corrected and uncorrected thresholds in the VBM statistical analysis, only the corrected results were selected to avoid false positive findings.

**Data extraction**

The data on demographic and clinical characteristics, methodological features, and coordinates information, and VBM results were extracted from each included study. Two authors independently extracted the data from the studies according to a predefined and standardized data extraction form. Any inconsistencies were discussed until an agreement was reached.

**Coordinate-based meta-analysis (CBMA) of** **VBM studies**

This present CBMA was performed using SDM-PSI version 6.21 ([www.sdmproject.com](http://www.sdmproject.com)). Details of the SDM-PSI method have been published previously [3, 4]. Opposed to current CBMA methods, SDM-PSI made significant algorithm improvements that used standard voxelwise tests and, importantly, conducted a standard permutation of subject images (PSI). In addition, SDM-PSI used unbiased estimation of effect sizes based on MetaNSUE algorithms, random-effects models, Freedman-Lane-based permutations, and threshold-free cluster enhancement (TFCE) statistics, which made the CBMA results more accurate [3]. The meta-analytic processes were consistent with the standard SDM guidelines [4] and were briefly summarized here: (1) peak coordinates of significant GM differences between migraineurs and healthy controls were selected and organized according to the SDM-PSI inclusion criteria; (2) SDM-PSI estimated the lower and upper bounds of possible effect size images for each study within a GM mask using an a 20 mm full width half maximum (FWHM) anisotropic Gaussian kernel and 2 mm voxel size; (3) SDM used MetaNSUE based on multiple imputations of maximum likelihood estimation (MLE) to estimate the most likely effect size and its standard error [5]; (4) each imputed dataset was meta-analyzed and then Rubin's rules were used to combine these imputed meta-analyzed datasets [5]; (5) SDM-PSI recreated subject images and then conducted a standard PSI, in which the maximum statistic of the combined meta-analysis image is saved. In order to allow family-wise error rate correction (FWER) for multiple comparisons, the distribution of the maximum statistic was used. (6) Hedge’s g-corrected effect sizes were calculated at the group level. (7) A random-effects model was used for the meta-analysis, in which the design matrix included any covariate used in the MLE step and the weight of a study was the inverse of the sum of its variance and the between-study heterogeneity τ^2^.

Finally, voxel-wise results were reported using the recommended thresholds: threshold-free cluster enhancement (TFCE)-based FWER p < 0.05, voxels extent ≥ 10 [3, 4].

**Sensitivity analysis**

Sensitivity analysis was performed to check the stability of meta-analytic findings by iteratively repeating the same analysis K-1 (where K is the number of the datasets) times, excluding one dataset each time [6, 7].

**Analyses of heterogeneity and publication bias**

If the CBMA reported significant clusters, the values from peak coordinates were extracted for the following supplemental analyses: heterogeneity and publication bias analyses. In order to estimate between-study variability in the results, a heterogeneity analysis was performed using a random effects model with Q statistics. We examined the presence of publication biases by visual inspection of funnel plots and further Egger’s weighted regression test. An asymmetric plot and p < 0.05 were deemed significant, respectively.

**Subgroup meta-analyses**

The following subgroup meta-analyses were conducted, including the datasets comprising patients only with episodic migraine and only with chronic migraine, the datasets comprising patients only with migraine without aura and only with aura, the datasets acquiring images with a 3.0T MRI scanner, the datasets employing statistical parametric mapping (SPM) software, version 8 or 12, the datasets exploring GM volume differences, the datasets using a smoothing kernel of 8 mm, the datasets treating age, gender, and intracranial volume (ICV) as covariates of no interest in the VBM statistical model, the datasets applying corrected thresholds for multiple comparisons, and the datasets including more than 50 participants in the patient group.

**Meta-regression analyses**

Meta-regression analyses were performed to examine the potential influences of moderator variables, such as mean age and female percentage of the patient sample, illness duration, and headache attack frequency per month on GM abnormalities if they were reported in at least ten datasets. Statistical significance was determined using p < 0.05 TFCE-FWER corrected for multiple comparisons and cluster size ≥ 10 voxels.

**Supplementary Results**

**Study inclusion and characteristics**

According to the search strategy, 1023 records were identified for consideration, of which 991 were excluded based on the study selection criteria. Finally, 32 studies were eligible to be included in the CBMA. These VBM studies included 41 datasets involving a total of 1252 patients with migraine (988 females/264males, mean age 37.63 years) and 1025 healthy controls (794 females/231males, mean age 36.78 years). Supplementary Table 1 is a summary of the demographic and clinical characteristics and VBM results of the included studies included in the meta-analysis. Supplementary Table 2 summarizes the imaging characteristics of VBM studies included in the meta-analysis.

**Meta-analysis**

CBMA of all VBM datasets using SDM-PSI found no consistent results regarding GM alterations in patients with migraine relative to healthy controls (TFCE-FWER, p < 0.05 and cluster size ≥ 10 voxels).

**Sensitivity analysis**

Sensitivity analysis showed that the negative finding was preserved throughout all 41 combinations of datasets.

**Analyses of heterogeneity and publication bias**

We could not perform heterogeneity and publication bias analyses because there were no significant clusters reported in the CBMA.

**Subgroup meta-analyses**

The findings of all subgroup meta-analyses were consistent with the results of the main CBMA of all included datasets, including subgroup meta-analyses of those datasets comprising patients only with episodic migraine (n = 20) and only with chronic migraine (n = 6), the datasets comprising patients with migraine without aura (n = 21) and with aura (n = 6), the datasets acquiring images with a 3.0T MRI scanner (n = 32), the datasets employing statistical parametric mapping (SPM) software, version 8 or 12 (n = 32), the datasets exploring GM volume differences (n = 36), the datasets using a smoothing kernel of 8 mm (n = 18), the datasets treating age, gender, and intracranial volume (ICV) as covariates of no interest in the VBM statistical model (n = 10), the datasets applying corrected thresholds for multiple comparisons (n = 36), and the datasets including more than 50 participants in the patient group (n = 7).

**Meta-regression analyses**

Meta-regression analyses showed that variables such as mean age (n = 41) and female percentage (n = 41) of the patient sample, illness duration (n = 33), and headache attack frequency per month (n = 36) did not correlate with GM volumes (p < 0.05 TFCE-FWER corrected and cluster size ≥ 10 voxels).

**References**

1. Muller VI, Cieslik EC, Laird AR et al. (2018) Ten simple rules for neuroimaging meta-analysis. Neurosci Biobehav Rev.84:151-61. doi:10.1016/j.neubiorev.2017.11.012.

2. Tahmasian M, Sepehry AA, Samea F et al. (2019) Practical recommendations to conduct a neuroimaging meta-analysis for neuropsychiatric disorders. Human brain mapping.40(17):5142-54. doi:10.1002/hbm.24746.

3. Albajes-Eizagirre A, Solanes A, Vieta E et al. (2019) Voxel-based meta-analysis via permutation of subject images (PSI): Theory and implementation for SDM. NeuroImage.186:174-84. doi:10.1016/j.neuroimage.2018.10.077.

4. Albajes-Eizagirre A, Solanes A, Fullana MA et al. (2019) Meta-analysis of Voxel-Based Neuroimaging Studies using Seed-based d Mapping with Permutation of Subject Images (SDM-PSI). Journal of visualized experiments : JoVE.(153). doi:10.3791/59841.

5. Albajes-Eizagirre A, Solanes A, Radua J. (2019) Meta-analysis of non-statistically significant unreported effects. Statistical methods in medical research.28(12):3741-54. doi:10.1177/0962280218811349.

6. Radua J, Mataix-Cols D. (2009) Voxel-wise meta-analysis of grey matter changes in obsessive-compulsive disorder. Br J Psychiatry.195(5):393-402. doi:10.1192/bjp.bp.108.055046.

7. Radua J, Rubia K, Canales-Rodriguez EJ et al. (2014) Anisotropic kernels for coordinate-based meta-analyses of neuroimaging studies. Front Psychiatry.5:13. doi:10.3389/fpsyt.2014.00013.

**Supplementary Table 1.** **Demographic and clinical characteristics and VBM results of the studies included in the meta-analysis**

| Study | Sample (female) | Age, years (SD) | WoA/WA | Attack frequency/month (SD) | Migraine type | Duration, years (SD) | Results |
| --- | --- | --- | --- | --- | --- | --- | --- |
| Arkink et al., 2017 | Patients^1^ 19 (18)  Patients^2^ 14 (13)  Controls 48 (30) | 47 (8)  47 (9)  47 (12) | 19/0  0/14 | 2.8 (1.9)  2.5 (1.2) | EM | 29 (15)  28 (10) | Decreased GMV in right lingual gyrus in migraine patients without aura  No change in migraine patients with aura |
| Bonanno et al., 2020 | Patients^1^ 14 (14)  Patients^2^ 14 (14)  Controls 14 (14) | 43.5 (3.25)  42.36 (2.95)  42.5 (5.17) | 14/0  0/14 | 1.89 (1.18)  2.48 (1.40) | EM | 6.78 (3.66)  5.21 (1.31) | Increased GMV in right thalamus and decreased GMV in cerebellum, left cerebellum crus 1, left superior/medial frontal gyrus, right inferior/middle frontal gyrus, right superior frontal gyrus, left fusiform gyrus, left Brodmann area 20, right parahippocampal gyrus, and right insula in migraine patients without aura  Increased GMV in right superior parietal gyrus and left thalamus and decreased GMV in right cerebellum, left postcentral gyrus, right inferior frontal gyrus, left Brodmann area 22, and left lingual gyrus in migraine patients with aura |
| Celle et al., 2018 | Patients 25 (19)  Controls 39 (30) | 75 (1.2)  75.4 (0.9) | 19/6 | 7.4 (3.4) | EM | 46.2 (16.4) | No change |
| Chanraud et al., 2014 | Patients 18 (13)  Controls 17 (13) | 45.7 (10.2)  46.5 (10.5) | NA/NA | NA | EM | 25.9 (14.8) | No change |
| Chen et al., 2018 | Patients 56 (37)  Controls 43 (28) | 37.5 (7.6)  36.2 (7.7) | 56/0 | 13.8 (10.5) | 31 EM/25 CM | 16.2 (9.7) | No change |
| Coppola et al., 2015 | Patients^3^ 14 (11)  Patients^4^ 10 (8)  Controls 15 (11) | 31.6 (7.6)  33.3 (12.1)  28.6 (4) | 14/0  10/0 | 3.4 (2.4)  4 (3.4) | EM  EM | 16.5 (6.6)  13.1 (9.9) | Decreased GMD in right inferior parietal lobule, inferior temporal gyrus, and superior temporal gyrus and left temporal pole in migraine patients scanned between attacks vs. controls  Increased GMD in right lenticular nuclei, left temporal pole, and bilateral insula in migraine patients scanned ictally vs. controls |
| Coppola et al., 2017 | Patients 20 (14)  Controls 20 (13) | 31.3 (10.2)  28.5 (4.1) | 20/0 | 23 (6.8) | CM | 15 (13.1) | No change |
| Hougaard et al., 2016 | Patients 60 (42)  Controls 60 (42) | 33.36 (10.3)  33.39 (10.3) | 0/60 | NA | NA | NA | No change |
| Hubbard et al., 2014 | Patients 17 (13)  Controls 18 (14) | 41.71 (12.2)  38.89 (11.25) | NA/NA | 11.65 (10.07) | 4 EM/13 CM | 12.53 (8.41) | Increased GMV in left hippocampus |
| Kim et al., 2008 | Patients 20 (17)  Controls 33 (29) | 33.7 (11.3)  33.8 (10.5) | 15/5 | 2.73 (0.91) | EM | 9.8 (6) | Decreased GMV in bilateral insula, motor/premotor cortex, and prefrontal cortex, left dorsal anterior cingulate cortex, right dorsal posterior cingulate cortex, right inferior and superior parietal cortex, orbitofrontal cortex, and visual cortex |
| Lai et al., 2016 | Patients^5^ 33 (27)  Patients^6^ 33 (27)  Controls 33 (27) | 39.7 (10.7)  40.2 (10)  39.7 (11.1) | 33/0  33/0 | 19.5 (9.8)  23.2 (8.9) | CM  CM | 16.1 (10.6)  18.4 (10.4) | Decreased GMV in right inferior frontal gyrus and temporal pole/fusiform gyrus/ inferior temporal gyrus and left temporal pole/fusiform gyrus and precentral gyrus in chronic migraine without medication overuse  Decreased GMV in bilateral rectal gyrus/nucleus accumbens and inferior frontal gyrus, right frontal operculum, middle frontal gyrus, precuneus, and lingual gyrus, and left superior frontal gyrus, precentral gyrus, superior occipital gyrus/cuneus, and cerebellum in chronic migraine with medication overuse |
| Lai et al., 2020 | Patients 30 (23)  Controls 30 (22) | 33.2 (9.8)  32.4 (8.3) | NA | 16.5 (8.3) | CM | 13.2 (8.8) | No change |
| Linnman et al., 2018 | Patients 9 (5)  Controls 9 (6) | 25.9 (4)  25.6 (4) | 9/3 | 5.2 (3) | EM | 13.1 (9) | Decreased GMD in middle and anterior cingulate gyrus, bilateral insular cortices, left inferior temporal gyrus, right cerebellum and left precentral gyrus |
| Lisicki et al., 2018 | Patients 20 (16)  Controls 20 (15) | 32.2 (12.8)  34.8 (11.3) | 20/0 | 4.1 (2.6) | EM | NA | No change |
| Liu et al., 2015 | Patients 135 (135)  Controls 111 (111) | 21.7 (2.1)  21.3 (0.90) | 135/0 | 6.4 (5) | EM | 5.6 (2.9) | Decreased GMV in middle frontal gyrus, superior frontal gyrus, inferior parietal lobule, supramarginal gyrus, temporal cortices, and occipital cortices and increased GMV in hippocampus, parahippocampal gyrus amygdala, cerebellum, and occipital cortices |
| Liu et al., 2020 | Patients 56 (43)  Controls 37 (27) | 40.3 (10.5)  39.4 (9.3) | 47/9 | 19.2 (7.1) | 12EM/44CM | 17.2 (11.3) | Decreased GMV in right supramarginal gyrus and increased GMV in right cerebellum crus II |
| Masson., et al 2020 | Patients 19 (13)  Controls 19 (13) | 32.7 (8.7)  33.6 (11.5) | 19/0 | 3.3 (1.1) | EM | 16.8 (7.4) | No change |
| Matharu et al., 2003 | Patients^6^ 11 (10)  Controls 11 (10)  Patients^5^ 17 (16)  Controls 17 (16) | 31 (7.3)  31 (7.3)  34 (8.3)  34 (8.3) | 0/11  17/0 | NA  NA | NA  NA | NA  NA | No change in migraine patients with aura  No change in migraine patients without aura |
| Mehnert and May, 2017 | Patients 54 (45)  Controls 54 (45) | 34.3 (11.9)  32.6 (11.50 | 40/14 | 11.8 (5.4) | 8 EM/46 CM | 18.1 (12.3) | Decreased GMV in bilateral precentral gyrus, right postcentral gyrus, left supramarginal, and a part of the left angular gyrus and increased GMV in right temporal, occipital, and cerebellar areas and left angular gyrus and pallidum. |
| Messina et al., 2018 | Patients 73 (50)  Controls 46 (29) | 35.1 (4.3)  32.9 (4.3) | NA/NA | 3.5 (1.1) | EM | 15 (3.3) | No change |
| Neeb et al., 2017 | Patients^7^ 21 (15)  Patients^8^ 21 (15)  Controls 21 (15) | 49.04 (7.46)  49.36 (7.62)  49.4 (7.79) | 21/0  21/0 | 15.9 (2.95)  5.33 (1.59) | CM  EM | 24.43 (8.3)  26.71 (14.42) | Increased GMV in right amygdala and putamen in CM  No change in EM |
| Niddam et al., 2016 | Patients^5^ 26 (17)  Patients^6^ 26 (17)  Controls 26 (17) | 32.3 (9.8)  28.3 (7.5)  31.2 (5.8) | 26/0  0/26 | 2.6 (1.2)  2.4 (2.1) | EM  EM | 13.5 (8)  13.1 (7.8) | No change in migraine patients without aura  Decreased GMV in right parahippocampus in migraine patients with aura |
| Palm-Meinders et al., 2017 | Patients 84 (57)  Controls 35 (25) | 57.8 (8)  54.6 (7.8) | 32/52 | 0.75 (0.25) | NA | NA | No change |
| Rocca et al., 2006 | Patients 16 (15)  Controls 15 (13) | 42.7 (7.5)  38.6 (6.5) | 9/7 | 1.7 (4.8) | NA | 24.8 (11.5) | Decreased GMD in bilateral anterior cingulated gyrus and middle frontal gyrus, right superior frontal gyrus, precentral gyrus, and middle temporal gyrus, and left inferior temporal gyrus and uncus |
| Schmidt-Wilcke et al., 2008 | Patients 35 (32)  Controls 31 (31) | 32.4 (9.2)  32.3 (12.6) | 35/0 | NA | 32 EM/3 CM | NA | No change |
| Schmitz et al., 2008 | Patients 28 (28)  Controls 28 (28) | 43.5 (8.21)  42.5 (9.31) | 20/8 | 3.5 (1.97) | NA | 30.5 (11.43) | Decreased GMD in superior frontal gyrus |
| Tedeschi et al., 2016 | Patients^5^ 20 (12)  Patients^6^ 20 (12)  Controls 20 (12) | 30.05 (6.9)  30.1 (7.4)  29.15 (5.8) | 20/0  0/20 | 6.65 (3.2)  0.74 (0.63) | EM  EM | 11.2 (7.8)  10.96 (8.6) | No change  No change |
| Wang et al., 2019 | Patients 21 (17)  Controls 21 (16) | 36.81 (11.61)  36.15 (12.11) | 21/0 | 1.86 (1.2) | NA | 11.57 (7.32) | Decreased GMV in right supplementary motion area |
| Wei et al., 2019 | Patients 33 (28)  Controls 22 (17) | 36.06 (10.58)  32.86 (7.2) | 33/0 | 3 (0.25) | EM | 8 (2.8) | No change |
| Yang et al., 2019 | Patients 22 (18)  Controls 45 (33) | 36.8 (9.9)  40.6 (10.7) | 18/4 | 8.9 (4.8) | NA | 6.7 (7.8) | Decreased GMV in left lateral occipital cortex, cerebellum vermis, and cerebellum and increased GMV in right frontal pole and middle frontal gyrus |
| Yu et al., 2020 | Patients^8^ 39 (30)  Patients^7^ 17 (8)  Controls 35 (20) | 39.74 (11.59)  49.59 (14.64)  34.91 (10.89) | 39/0  17/0 | 3.75 (2.64)  19.56 (4.17) | EM  CM | NA  NA | Decreased GMV in bilateral anterior cingulate cortex and middle frontal gyrus and increased GMV in bilateral periaqueductal grey matter and left hippocampus/parahippocampal gyrus in EM  Decreased GMV in bilateral anterior cingulate cortex and left hippocampus/parahippocampal gyrus and increased GMV in bilateral periaqueductal grey matter, right middle frontal gyrus, and left superior frontal gyrus in CM |
| Zhang et al., 2017 | Patients 32 (24)  Controls 32 (24) | 38.3 (10.16)  38.8 (10.02) | 32/0 | 3.36 (2.55) | EM | 9.5 (6.23) | Increased GMV in bilateral cerebellar culmen (lobule I-IV and lobule V) extending to the lingual gyrus, thalamus, fusiform and parahippocampa gyrus |

SD, standard deviation; WoA, migraine patients without aura; WA, migraine patients with aura; controls, headache-free controls; EM, episodic Migraine; GMV, gray matter volume; NA, not available; CM, chronic migraine; GMD, gray matter density; 1, migraine patients without aura; 2, migraine patients with aura; 3, patients without aura scanned between the attacks; 4, patients without aura scanned during the attacks; 5, patients with chronic migraine without medication overuse; 6, patients with chronic migraine with medication overuse; 7, patients with chronic migraine; 8, patients with episodic migraine

**Supplementary Table 2.** **Imaging characteristics of VBM studies included in the meta-analysis**

| Study | MR Scanner | Coil channels | MR sequence | Slice thickness (mm) | Software | Pipeline | Modulation | FWHM (mm) | Covariate | Statistics* |
| --- | --- | --- | --- | --- | --- | --- | --- | --- | --- | --- |
| Arkink et al., 2017 | Philips, 1.5 T | NA | TFE | 1 | SPM8 | VBM-DARTEL | Yes | 8 | age, sex, total parenchymal volume | FWE, p < 0.05 |
| Bonanno et al., 2020 | Philips, 3.0 T | 32-channel | NA | 1 | SPM12 | CAT12 VBM-DARTEL | Yes | 8 | age, attacks per year, frequency of attack, duration of headache attacks and TIV | FWE, p < 0.05 |
| Celle et al., 2018 | Siemen, 1.5 T | NA | MP-RAGE | 1 | SPM8 | VBM-DARTEL | Yes | default | NA | FWE, p < 0.05 |
| Chanraud et al., 2014 | Philips, 3 T | quadrature head coil | MP-RAGE | 1 | NA | optimized VBM | Yes | NA | TIV | FDR, p < 0.05 |
| Chen et al., 2018 | Siemens, 3 T | 32-channel | MP-RAGE | 1 | SPM8 | VBM8-DARTEL | No | 8 | age, sex, BDI | FWE, p < 0.05 |
| Coppola et al., 2015 | Siemens, 3 T | NA | NA | 1 | SPM8 | VBM8 | Yes | 8 and 12 | NA | uncorrected, p < 0.001 |
| Coppola et al., 2017 | Siemens, 3 T | NA | MP-RAGE | 1 | SPM12 | CAT12 VBM-DARTEL | Yes | 8 | NA | FWE, p < 0.05 |
| Hougaard et al., 2016 | Philips, 3 T | 32-channel | TFE | 1 | FSL | optimized VBM | Yes | 3 | age, gender, illness duration, attack frequency | TFCE |
| Hubbard et al., 2014 | Siemens, 3 T | 12-channel | MP-RAGE | 1 | SPM8 | VBM8-DARTEL | Yes | 8 | age, TIV | GRF, p < 0.05 |
| Kim et al., 2008 | Siemens, 1.5 T | NA | MP-RAGE | 1.25 | SPM2 | optimized VBM | Yes | 8 and 10 | age, sex, TIV | uncorrected, p < 0.001 |
| Lai et al., 2016 | GE, 1.5 T | 8-channel | IR-FSPGR | 1.5 | SPM8 | VBM-DARTEL | Yes | 8 | age, migraine ictal status | FWE, p < 0.05 |
| Lai et al., 2020 | Siemens, 3 T | 32-channel | MP-RAGE | 1 | SPM12 | VBM-DARTEL | Yes | 4 | age, sex, TIV | FDR, p < 0.05 |
| Linnman et al., 2018 | Siemens, 3 T | 8-channel | MP-RAGE | 1 | SPM12 | VBM-DARTEL | No | 8 | age, sex | FWE, p < 0.05 |
| Lisicki et al., 2018 | Siemens, 3 T | NA | 3D-gradient echo | 1.2 | SPM12 | VBM-DARTEL | Yes | 6 | whole brain size | FWE, p < 0.05 |
| Liu et al., 2015 | GE, 3 T | 8-channel | FSPGR | 1 | FSL 4 .1 | optimized VBM | Yes | 4 | NA | FWE, p < 0.05 |
| Liu et al., 2020 | GE, 3 T | 8-channel | IR-FSPGR | 1 | SPM12 | VBM-DARTEL | Yes | 8 | age, sex, TIV, and HADS scores | FWE, p < 0.05 |
| Masson., et al 2020 | Siemens, 3T | 64-channel | MP-RAGE | 0.9 | SPM12 | VBM-DARTEL | Yes | 10 | age, sex, TIV | TFCE-FWE, p < 0.05 |
| Matharu et al., 2003 | Siemens, 2 T | NA | MP-RAGE | 1.5 | SPM99 | optimized VBM | Yes | 10 | global mean voxel value | GRF, p < 0.05 |
| Mehnert and May, 2017 | Siemens, 3 T | 32-channel | MP-RAGE | 1 | SPM12 | CAT12, DARTEL | Yes | 8 | age, sex, TIV | uncorrected, p < 0.001 |
| Messina et al., 2018 | Philips, 3 T | NA | fast field echo | 0.8 | FSL/SIENAX and SPM12 | VBM-DARTEL | Yes | 8 | age, sex, TIV | FDR, p < 0.05 |
| Neeb et al., 2017 | Siemens, 3 T | 32-channel | MP-RAGE | 1 | SPM8 | VBM8-DARTEL | Yes | 10 | age, sex, TIV | FWE, p < 0.05 |
| Niddam et al., 2016 | GE, 3 T | NA | FSPGR | 1 | SPM8 | optimized VBM | Yes | 8 | age, TIV | FDR, p < 0.05 |
| Palm-Meinders et al., 2017 | Philips, 1.5 T | NA | fast field echo | 1 | SPM8 | VBM-DARTEL | Yes | 8 | NA | FWE, p < 0.05 |
| Rocca et al., 2006 | Philips, 3 T | NA | MP-RAGE | 1 | SPM2 | optimized VBM | Yes | 12 and 8 | NA | uncorrected, p < 0.001 |
| Schmidt-Wilcke et al., 2008 | Siemens, 1.5 T | NA | MP-RAGE | NA | SPM2 | optimized VBM | Yes | 12 | NA | p < 0.05, corrected |
| Schmitz et al., 2008 | Philips, 3 T | NA | NA | 1.2 | SPM2 | optimized VBM | No | 8 and 10 | NA | FWE, p < 0.05 |
| Tedeschi et al., 2016 | GE, 3 T | 8-channel | IR-FAFSPGR | 1.2 | FSL/SIENAX and SPM8 | VBM8-DARTEL | Yes | 8 | age, sex, TIV | FWE, p < 0.05 |
| Wang et al., 2019 | GE, 3 T | 8-channel | MP-RAGE | 1 | SPM8 | VBM8-DARTEL | Yes | 8 | NA | p < 0.05, uncorrected |
| Wei et al., 2019 | Philips, 3 T | 8-channel | NA | 1 | SPM8 | VBM-DARTEL | Yes | 8 | NA | FDR, p < 0.05 |
| Yang et al., 2019 | GE, 3 T | 8-channel | IR-FSPGR | 1 | SPM12 | CAT12, VBM-DARTEL | Yes | 8 | age, sex, TIV | FWE, p < 0.05 |
| Yu et al., 2020 | Siemens, 3 T | NA | FSPGR | 1 | SPM8 | VBM-DARTEL | Yes | 8 | age | AlphaSim, p < 0.05 |
| Zhang et al., 2017 | Siemens, 3 T | 12-channel | MP-RAGE | 1 | SPM12 | CAT12, VBM-DARTEL | Yes | 8 | TIV | FDR, p < 0.05 |

VBM, voxel-based morphometry; MR, magnetic resonance; FWHM, full width half maximum; NA, not available; TFE, turbo field echo; MP-RAGE, magnetization prepared rapid gradient echo; SPM, statistical parametric mapping; DARTEL, diffeomorphic anatomical registration through an exponentiated lie algebra; FWE, family-wise error; TIV, total intracranial volume; FDR, false discovery rate; BDI, beck depression inventory; CAT, computational anatomy toolbox; FSL, functional MRI of the brain software libraries package; HADS; Hospital Anxiety and Depression Scale; TFCE, threshold-free cluster enhancement; GRF, gaussian fandom field theory; IR-FAFSPGR, inversion-recovery fluid-attenuated fast spoiled gradient-recalled echo; FSPGR, fast spoiled gradient recalled sequence; SIENAX, structural image evaluation with normalisation of atrophy cross-sectional; *, When both corrected and uncorrected thresholds are applied in the VBM statistical analysis, the results of the former analysis was selected to avoid false positive findings.
